# A Central Role for Thiols in Plant Tolerance to Abiotic Stress

**DOI:** 10.3390/ijms14047405

**Published:** 2013-04-02

**Authors:** Lyuben Zagorchev, Charlotte E. Seal, Ilse Kranner, Mariela Odjakova

**Affiliations:** 1 Department of Biochemistry, Faculty of Biology, Sofia University, 1164 Sofia, Bulgaria; E-Mail: lzagortchev@yahoo.co.uk; 2 Seed Conservation Department, Royal Botanic Gardens Kew, Wakehurst Place, Ardingly, West Sussex, RH17 6TN, UK; E-Mail: c.seal@kew.org; 3 Institute of Botany, University of Innsbruck, Sternwartestraße 15, A-6020 Innsbruck, Austria; E-Mail: ilse.kranner@uibk.ac.at

**Keywords:** cysteine, glutaredoxin, glutathione, glutathionylation, phytochelatins, reactive oxygen species, redox state, signalling, sulphur metabolism, thioredoxin

## Abstract

Abiotic stress poses major problems to agriculture and increasing efforts are being made to understand plant stress response and tolerance mechanisms and to develop new tools that underpin successful agriculture. However, the molecular mechanisms of plant stress tolerance are not fully understood, and the data available is incomplete and sometimes contradictory. Here, we review the significance of protein and non-protein thiol compounds in relation to plant tolerance of abiotic stress. First, the roles of the amino acids cysteine and methionine, are discussed, followed by an extensive discussion of the low-molecular-weight tripeptide, thiol glutathione, which plays a central part in plant stress response and oxidative signalling and of glutathione-related enzymes, including those involved in the biosynthesis of non-protein thiol compounds. Special attention is given to the glutathione redox state, to phytochelatins and to the role of glutathione in the regulation of the cell cycle. The protein thiol section focuses on glutaredoxins and thioredoxins, proteins with oxidoreductase activity, which are involved in protein glutathionylation. The review concludes with a brief overview of and future perspectives for the involvement of plant thiols in abiotic stress tolerance.

## 1. Introduction

The understanding of the mechanisms of plant stress tolerance is key to the successful selection of tolerant crops in growth areas affected by environmental pollution and climate change, which is predicted to increase drought and salinisation. Recently, a number of “omics” studies, including transcriptional regulation of stress-related genes, were conducted [1,2], and proteomic studies have identified proteins with significance to plant stress response [3,4]. However, the data still do not provide sufficient information on the particular mechanisms that underpin the stress phenome. Low-molecular-weight compounds were also found to be a key component of the plant stress response. From the 1980ies onwards [5–7], much research into biochemical stress markers was targeted to elucidate components of oxidative stress, and metabolomic studies of plant stress tolerance are now beginning to reveal a more complete picture [8].

Many of the mechanisms of abiotic stress response in plants were found to be similar, if not identical, independent of the stress factor [9]. Such non-specific stress responses may explain the cross-tolerance to various stresses of a particular resistant species or crop line. It has been established that temperature stress [10], heavy metals [11], salt stress and water deficit [12] can all lead to increased production of reactive oxygen species (ROS) with downstream alterations of oxidative signalling. Evidence is emerging that a number of non-protein and protein thiols, together with a network of sulphur-containing molecules and related compounds, also fundamentally contribute to plant stress tolerance [13,14].

**Figure 1:**
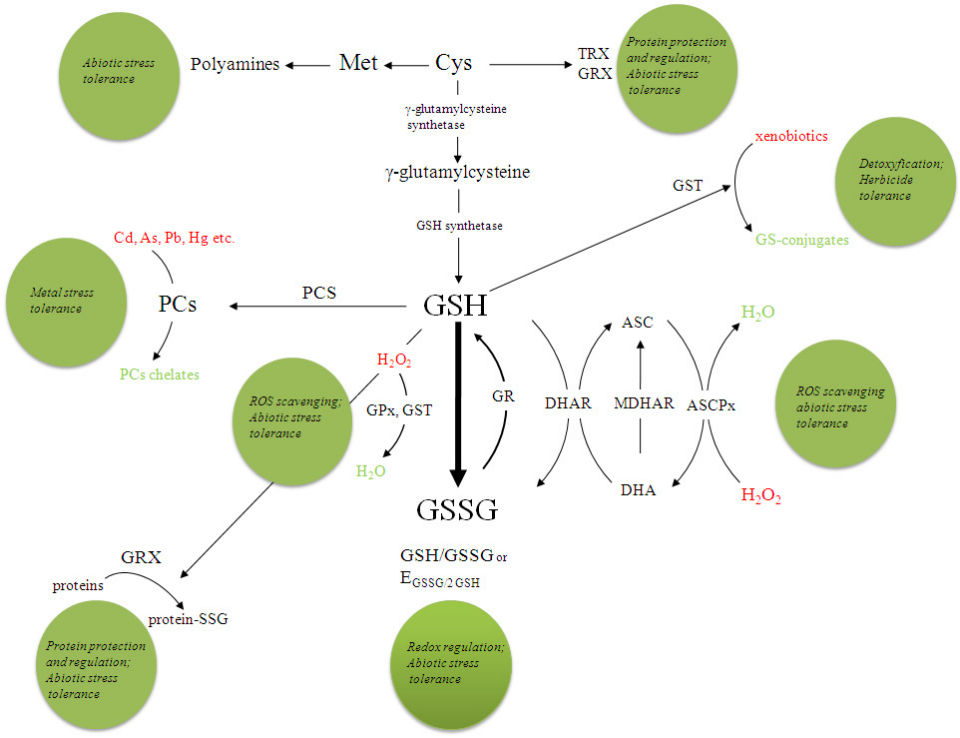
Overview of the roles of thiols in plant tolerance to abiotic stress. Potential roles of and the significance for abiotic stress tolerance are depicted by green circles next to SH-adducts. Potential deleterious compounds are shown in red and their adducts in green font. The dotted circle represents the half-cell reduction potential of the glutathione disulphide (GSSG)/2 glutathione (GSH) redox couple. Met, methionine; Cys, cysteine; TRX, thioredoxin; GRX, glutaredoxin; PC, phytochelatins; PCS, phytochelatin synthetase; GST, glutathione-S-transferase; GPx, glutathione peroxidase; GR, glutathione reductase; DHAR, dehydroascorbate reductase; ASC, ascorbate; MDHAR, monodehydroascorbate reductase; DHA, dehydroascorbate; ASCPx, ascorbate peroxidase.

The aim of this review is to provide an update on the most important non-protein and protein thiols with respect to plant tolerance to abiotic stress, which are summarised in Figure 1. The sulphur-containing amino acids, cysteine (Cys), the primary product of sulphate assimilation, and methionine (Met) are discussed first, followed by a detailed discussion of the roles of the most abundant low-molecular-weight thiol, the tripeptide glutathione (γ-glutamyl-cysteinyl-glycine; GSH), which is synthesised from Cys and the various protective mechanisms in which GSH and glutathione disulphide (GSSG) are involved in. Glutathione is a key water-soluble antioxidant and plays a central part in ROS scavenging through the GSH-ascorbate cycle and as an electron donor to glutathione peroxidase (GPx). It is the storage form and the long-distance transport form of reduced sulphur, is involved in the detoxification of heavy metals and xenobiotics [15] and in the regulation of the cell cycle. Lastly, protein thiols, which include thioredoxins and glutaredoxins and the glutathionylation of protein sulphydryl groups, are considered as protective and regulatory mechanisms. Despite recent progress, our knowledge of the biochemical interactions between low- and high-molecular-weight thiols and other molecules is still incomplete. However, the data available so far suggest that thiols play a central role in the abiotic stress tolerance of plants.

## 2. Sulphur-Containing Amino Acids

### 2.1. Cysteine Biosynthesis and Free Cysteine Accumulation

Cys is the final product of sulphate assimilation, the process by which sulphur is taken up by plants. Hence, Cys is a central metabolite that serves as a sulphur donor for the synthesis of Met, iron-sulphur clusters, some vitamins, such as thiamine and biotin, of lipoic acid and coenzyme A, GSH and thiol-containing proteins [16]. Cys is synthesised in two steps. First, o-acetylserine is formed from Ser and acetyl-CoA, catalysed by serine acetyltransferase (SAT; EC 2.3.1.30), and then, reduced sulphur in the form of H_2_S is included by o-acetylserine (thiol) lyase (EC 4.2.99.8) by elimination of the acetate residue to form Cys. These two enzymes form the hetero-oligomeric Cys synthetase complex. There is a variety of Cys synthetase isoforms in protein-synthesising organelles, e.g., chloroplasts, mitochondria, and in the cytoplasm [17]. Using transgenic plants expressing or overexpressing different serine acetyltransferase (SAT) and O-acetylserine (thiol) lyase isoforms showed that the Cys synthetase complex is a primary enzyme of sulphur metabolism with significance to stress response. For example, the Cys synthetase complex appears to be involved in Cd tolerance in transgenic tobacco plants [18] and in H_2_S and SO_2_ toxicity resistance in transgenic poplar [19].

Unstressed cells contain low concentrations of free Cys, not more than 10–30 μM, but turnover rates are high [20]. The accumulation of free Cys is limited by feedback regulation of SAT, and expression of Cys-insensitive SATs is required for Cys accumulation [21]. An increase in free Cys levels in response to various abiotic stress factors has been reported [22,23]. In most studies, this increase was reported together with increased GSH concentrations, leading to the conclusion that Cys is mainly needed for the biosynthesis of sulphur-rich compounds with anti-stress activity, such as GSH and stress-related proteins. It is generally thought that at concentrations above 50 μM, Cys is toxic for plants [13]. Cys is a potent chelator of heavy metals ions, but Cys-metal ion complexes can trigger the Fenton reaction, thereby producing the highly toxic ^•^OH radical. Furthermore, free Cys is often irreversibly oxidised to different by-products [24]. Hence, the accumulation of free Cys can lead to undesirable loss of sulphur, which potentially compromises the stress coping mechanisms of the plant. It is uncertain whether reversible formation of cystine (cysteine-disulphide; CySS) is catalysed by cystine reductase (EC 1.8.1.6) in plants, as studies of this enzyme are rare [25] and no recent reports are available for plants. On the other hand, similar enzymes were found in different organisms, including protozoa [26] and bacteria [27].

The redox potential of the CySS/2Cys couple is regarded as an important biochemical marker for the early stages of various human diseases [28] and as an important antioxidant system and regulator of the redox state in parasites [29], and the CySS /2Cys redox state may have important roles in plant stress response, too [30]. The pathways of CySS metabolism in plant cells are still unclear; Olm *et al.*[31] suggested that molecules such as GSH, and enzymes, such as glutathione reductase (GR), thioredoxin reductase and glutaredoxins (GRXs), could be responsible for CySS reduction. In protozoa, thioredoxin reductase has such a function [32], but several studies of glutathione reductase (GR) showed that this enzyme is not capable of reducing CySS [33]. There are large gaps in our knowledge of Cys and CySS metabolism in plants, and closing them will further enhance our understanding of plant stress response.

### 2.2. Methionine

Similar to Cys, Met can undergo ROS-mediated oxidation to methionine sulfoxide (MetO) and this can lead to changes in protein conformation and activity [34]. The conversion of MetO to Met is mediated by Met sulfoxide reductases (MSRs; EC 1.8.4.11), a class of cytosolic and plastidic enzymes that are involved in ameliorating oxidative damage [35]. Unlike Cys, accumulation of free Met in plant cells under stress does not appear to be linked to antioxidant defence. There are few and ambiguous data on free Met accumulation under drought [36] and osmotic stress [37], whereby Met was not discriminated from valine in the latter report. Overexpression of cystathionine γ-synthase (EC 2.5.1.48) in tobacco [38] and ectopic expression of bacterial serine acetyltransferase (EC 2.3.1.30) in rice [39] led to increased levels of intracellular free Met, but these experiments were conducted with the aim to improve the nutritional qualities of the crops, considering that Met is also an essential amino acids for humans. Supplementation of the growth medium with Met improved stress survival of *E. coli*[40], but no similar *in vitro* experiments have been conducted with plant cultures.

Met is also a substrate for the synthesis of various polyamines with important roles in stress tolerance, the most prominent being putrescine, spermidine and spermine [41]. This biosynthetic pathway involves the intermediate S-adenosylmethionine (SAM) as a primary methyl donor, which further yields S-adenosyl-L-homocysteine that is metabolised to eventually regenerate Met. SAM is also a source for ethylene synthesis [42], reinforcing the pivotal role of Met in the plant stress response. Accumulation of polyamines, either free or conjugated, in response to abiotic stress and induction of the enzymes involved in their synthesis was reported in a number of case studies (reviewed in [41]). Similarly, transgenic plants expressing polyamine biosynthetic genes showed much higher stress tolerance than wild-type plants [43]. According to an extensive review by Groppa and Benavides [44], polyamines are involved in stress response and tolerance to almost every type of abiotic stress, such as salt, drought, chilling, UV, metal, ozone, wounding and, more generally, oxidative stress. Most importantly, polyamine biosynthesis does not consume sulphur and could be by-passed in a SAM independent biosynthesis; therefore, it can occur under sulphur-limited conditions [45].

## 3. Glutathione

### 3.1. GSH in Plant Stress Response

All plants contain GSH or GSH homologues, where the *C*-terminal amino acid glycine is replaced by another amino acid, such as β-alanine, serine or glutamate [15]. GSH is synthesised in two steps. First, γ-glutamyl-cysteine is formed in an ATP-dependent reaction, catalysed by glutamate–cysteine ligase, also known as γ-glutamyl-cysteine synthetase (EC 6.3.2.2), which is the rate-limiting reaction. Glutathione synthetase (EC 6.3.2.3) then catalyses the addition of glycine to γ-glutamyl-cysteine [15]. GSH synthesis can occur in the cytosol, chloroplasts and mitochondria [46], and both enzymes are encoded by single genes with alternate transcription start sites that are associated with their subcellular localisation [47]. Recently published results confirmed that the concentrations of GSH increased at least transiently in plants exposed to Cd [48], Pb [49], salt [30], drought [50] and heat stress [51].

Several studies of plants that overexpressed γ-glutamyl-cysteine synthetase or transgenic plants expressing bacterial γ-glutamyl-cysteine synthetase evaluated its effect on metal tolerance based on the assumption that higher levels of GSH and phytochelatins will lead to more efficient metal sequestration. Interestingly, several authors reported that increased expression of this enzyme may not be related to stress tolerance, especially Cd tolerance. Overexpression of γ-glutamyl-cysteine synthetase in *Arabidopsis*[52] and tomato [53] did not enhance resistance to Cd stress, despite increased levels of GSH and phytochelatins. Similarly, the expression of bacterial γ-glutamyl-cysteine synthetase in *Arabidopsis* did not enhance Cd tolerance and even caused Cd sensitivity [54], although some resistance to As and Hg was observed. Transgenic cottonwood also showed enhanced As tolerance [55]. Song *et al.*[56] showed that Cu stress lead to a two- to three-fold increase in the concentration of γ-glutamyl-cysteine synthetase in different rice varieties, with the sensitive genotype showing a greater increase. The overall conclusion was that Cd tolerance may rely on different factors than tolerance to other metals (see Section 3.5).

Although increased levels of GSH may be required for metal tolerance, it seems that tolerant genotypes tend to keep their γ-glutamyl-cysteine synthetase levels lower than sensitive ones. Similarly, long term water deficit in *Vigna radiata* caused a decrease in both γ-glutamyl-cysteine synthetase activity and its transcript levels [57] in roots and even lower activity, combined with higher mRNA levels during the recovery period. This is in disagreement with the assumption that abiotic stress tolerance involves an increase in γ-glutamyl-cysteine synthetase abundance and activity along with increases in Cys and GSH concentrations, as shown for salt stress [58]. Overall, in most, if not all, of the cited studies, glutathione synthetase expression and activity increased simultaneously with that of γ-glutamyl-cysteine synthetase. Loss of function of any of the two enzymes proved to be lethal to early developmental stages, and GSH deficiency resulted in increased sensitivity to Cd in *Arabidopsis*[59].

Homoglutathione (hGSH) is a homologue of GSH, characteristic for legumes in which the *C*-terminal Gly is substituted by β-Ala and, overall, has the same functions as GSH [15]. It is regarded as an important regulator of root nodule formation, symbiotic interactions and nitrogen fixation [60], serves functions in the transport of reduced sulphur and has antioxidant activity, as GSH does. Legumes are also capable of synthesising homophytochelatins in response to heavy metal stress [61]. A study of hGSH synthetase transcript levels and activity and hGSH concentration in root nodules exposed to different stresses [62] did not reveal any significant increase in hGSH synthetase, except for mRNA levels that rose upon Cd and H_2_O_2_ treatments. In contrast, Cruz de Carvalho *et al.*[63] found increased mRNA levels in the leaves of a drought tolerant cowpea cultivar, encoding hGSH synthetase during drought stress and desiccation, revealing different stress response patterns than in roots. The distribution of GSH and hGSH in different plant organs is species-specific and determined by the differential expression of the corresponding synthetase genes (summarised by [64]). Other authors reported that a glyphosate-resistant soybean line showed an increase in hGSH concentration upon glyphosate treatment, whereas in a glyphosate-sensitive line, it remained unchanged or decreased slightly; and, more importantly, the hGSH/hGSSG ratio remained higher in the resistant line [65]. The latter is an example of a complete overlap of hGSH and GSH functions, which is not surprising, as hGSH replaces GSH in soybean. A comparative study between soybean and white lupin subjected to Cd and As [66] showed that hGSH levels (in soybean) increased significantly more than GSH levels (in white lupin). Clemente *et al.*[67] also suggested that GSH and hGSH play distinct roles in plant development and stress response, based on the differential hormone-mediated expression and activity of GSH and hGSH synthetases. Hence, GSH and hGSH have largely the same functions, although in some cases, the two homologues may be differentially affected by stress in plant species that are able to synthesise both hGSH and GSH, but the importance of such effects is still to be elucidated.

### 3.2. Glutathione Redox State

GSH is the main cellular redox buffer that keeps the intracellular environment reduced. Typically, the GSH to GSSG ratio is about 20:1 in unstressed conditions, and this ratio shifts towards GSSG upon stress, as a result of ROS scavenging under conditions that compromise the reduction of GSSG to GSH [68]. This shift contributes to the signalling pathways that lead to programmed cell death, an important mechanism of stress resistance [69]. A reducing intracellular environment is needed to maintain protein structure and function (reviewed by [70]). A high GSH/GSSG ratio is supported by GR (EC 1.6.4.2), and stress-tolerant genotypes have higher ratios than stress-sensitive ones [71,72]. The GSH/GSSG ratio also varies with developmental stage. For example, a lower GSH/GSSG ratio is needed for somatic embryogenesis compared to the stage of cell proliferation [30].

Expressing the glutathione redox state through the glutathione half-cell reduction potential (E_GSSG/2GSH_) [73] takes into account the molar concentrations of GSH and GSSG instead of mere ratios. For concentration-dependent redox couples (note that two moles of GSH are required to produce one mole of GSSG), this is a critical consideration, as the concentration of GSH is important for cellular stress tolerance and viability. A clear correlation between E_GSSG/2GSH_ and cell viability has been demonstrated [74–76], and recently, the redox state of other low-molecular-weight thiols was also included to form a combined low-molecular-weight thiol-disulphide-based redox environment, E_thiol-disulphide/2 thiol_[77]. It is worth noting that the GSH intermediate, γ-glutamyl-cysteine, functionally replaced GSH, at least to some extent, at optimal conditions in a GSH synthetase loss of function mutant of cyanobacteria [78]. Elevated concentrations of γ-glutamyl-cysteine and its corresponding disulphide in plants could be deleterious, as there is no known enzyme capable of reducing it, and the only way for removal of the γ-glutamyl-cysteine disulphide is by degradation [79]. The concentration of Cys could also be of great importance, especially in conditions of abiotic stress [30]. Overall, the thiol-disulphide redox state serves as a marker of stress and cell viability, with important impacts upon the signalling mechanism during abiotic [80] and biotic stress [81].

ROS and redox signalling are in part transmitted through H_2_O_2_, and the best characterised pathway in plants is the “classic” chloroplast retrograde pathway [80]. The main regulator of redox state in chloroplasts is the plastoquinone pool, but shifts in the glutathione redox state and ascorbate pools were also correlated with development and stress response [82]. Redox signalling also likely includes an ascorbate-based redox shuttle across the plasma membrane that could regulate ascorbate concentrations in the different cell compartments, according to the specific requirements [83]. As an electron donor required for dehydroascorbate reduction, GSH could contribute to that system. The contribution of the GSH/GSSG couple to the fine tuning of the overall cellular redox state and its involvement in transcriptional control was proposed by Noctor and Foyer [7] and confirmed later [70].

### 3.3. The Ascorbate-Glutathione Cycle

In the ascorbate-glutathione cycle, also known as the Foyer-Halliwell-Asada cycle, superoxide can be converted to H_2_O_2_ by superoxide dismutase (SOD, EC 1.15.1.1). Then, H_2_O_2_ is further detoxified by ascorbate peroxidase (ASPx, EC 1.11.1.11), whereby monodehydroascorbate is produced, which can spontaneously dismutate to ascorbate and dehydroascorbate [82]. Monodehydroascorbate can also be reduced to ascorbate by NAD(P)H-dependent monodehydroascorbate reductase (MDHAR, EC 1.6.5.4) and dehydroascorbate reduced by GSH-dependent dehydroascorbate reductase (DHAR, EC 1.8.5.1). The latter is induced by various abiotic stresses and is thought to be critical for stress tolerance (see [84] and references therein). Abiotic stresses can simultaneously increase both the mRNA transcripts and activity of both enzymes [85], and overexpression of DHAR rather than MDHAR has been associated with stress tolerance [86]. However, a salt inducible MDHAR from the halophyte, *Avicennia marina*, may suffice to induce salt tolerance in transgenic plants [87]. Similar results were published for MDHAR from *Malpighia glabra* expressed in tobacco cells [88]. Evidence for the cooperation of various enzymes in the GSH-ascorbate cycle was demonstrated for transgenic tobacco plants, in which only those transformants showed increased tolerance to salt and cold stress that had DHAR and GR upregulated [89]. Therefore, the DHAR pathway may be less effective during sulphur starvation or in GSH-deficient mutants, but a shift from the GSH-dependent DHAR to GSH-independent MDHAR ascorbate regeneration under sulphur deficiency was not supported by experimental data. Contrary, MDHAR activity was shown to decrease in sulphur-starved plants [90].

Enhanced GSH-dependent dehydroascorbate reduction also improved tolerance to oxidative stress in DHAR transformants of *Arabidopsis*[91]. Interestingly, these transformants showed constitutively high DHAR activity, but also increased concentrations of both ascorbate and GSH compared to the wild-type. *E. coli*, expressing a tomato DHAR gene, also showed enhanced tolerance to H_2_O_2_, although mRNA levels in tomato plants slightly increased during cold treatment and decreased during treatment with abscisic acid (ABA) [92], and only mechanical wounding significantly increased the DHAR mRNA levels. An increase in the activity of two differentially expressed tomato DHARs was reported in response to salt and drought stress [93]. Gallie [94] concluded that non-GSH-dependent reduction of dehydroascorbate by MDHAR may be of greater importance for tolerance to at least some abiotic stresses, whereas GSH-dependent ascorbate regeneration is more important under optimal conditions, although more evidence is required before this hypothesis can be accepted.

A decrease in GR activity can affect the GSH/GSSG ratio, but also decrease the ascorbate pool and impact on ascorbate redox state [95] with an overall decrease in stress tolerance [96]. In turn, overexpression of GR [97] or increase in GR activity [98] has been related to stress tolerance. This enzyme mainly operates in the chloroplasts through cytosolic isoforms and mitochondrial isoforms are also known in higher plants. Increased GR activity was reported in response to various stresses, such as salt and Cd [summarised by 97], both in leaves and roots and in all subcellular fractions. As mentioned earlier [89], an increase in GR activity alone is not sufficient to confer stress tolerance. More likely, a coordinated and finely regulated action of all enzymes of the ascorbate-glutathione cycle in conjunction with that of other ROS-processing enzymes in all cell compartments is required for plant stress tolerance.

### 3.4. Glutathione-S-Transferases

Glutathione-S-transferases (GSTs, EC 2.5.1.18) comprise an extensive family of proteins with a great variety of functions. Up to 90 genes encoding GSTs are transcribed in different plant species, most of which are differentially induced by stress, and they play important parts in enzymatic thiol-dependent ROS scavenging mechanisms [99]. GSTs catalyse the conjugation of GSH to an electrophilic substrate [99], for example, they can catalyse the conversion of H_2_O_2_ at the expense of GSH, thereby producing GSSG. Known functions in plants include conjugation and sequestration of xenobiotics, transport of flavonoids, detoxification of ROS and organic radicals, programmed cell death under conditions of elevated ROS levels, signalling through flavonoids and participation in the fumarate synthesis during the tyrosine catabolism (reviewed by [100]).

Overexpression or heterologous expression of GSTs can contribute to abiotic stress tolerance. A chloroplastic GST from *Prosopis juliflora* improved drought stress tolerance in tobacco [101], and overexpression of GST in *Arabidopsis* increased salt tolerance [102]. A summary of recent experiments that support the role of GSTs in abiotic stress tolerance is presented in Table 1. GSTs are also good examples of genes from economically unimportant species that could be used for transformation of crops with enhanced stress tolerance. However, plant stress tolerance relies on an intricate network of various pathways, and changing only one part may produce unexpected results. For example, an *Arabidopsis* GST knockout mutant accumulated far more GSH than the wild-type and showed much higher tolerance to salt and drought stress [103]. In summary, GSTs comprise a large family of GSH-dependent enzymes that are involved in numerous stress-responsive mechanisms, mediating GSH functions in plant cells.

### 3.5. Phytochelatins

Phytochelatins are the best known metal ion chelators in plants and also in some fungi and invertebrates. They were first described in yeast [110] and cell cultures of *Rauvolfia serpentine*[111] about three decades ago. Since then, a number of reports confirmed their role in metal tolerance, and they received much attention in phytoremediation programmes [112]. Phytochelatins are oligomers of GSH characterised by the general structure (γ-Glu-Cys)*_n_*-Gly, whereby n varies between 2 and 11. The enzyme responsible for their synthesis is phytochelatin synthase, *i.e.*, γ-glutamyl-cysteine transpeptidase (EC 2.3.2.15), that is activated in the presence of metal ions. The phytochelatin complexes with ions are sequestered in the vacuole, thus ameliorating the toxic effects of metals. Numerous studies on phytochelatins are published every year, many of which report on the correlation between increased phytochelatins levels or heterologous expression of phytochelatin-related genes and metal tolerance (summarised for the year 2012 in Table 2). Phytochelatins most effectively chelate Cd, followed by As, whereas their efficiency to chelate other metals is not so conclusive. Sulphur deficient plants may switch to non-sulphur-based tolerance mechanisms, which often involve proline [24]. However, phytochelatins appear to be essential for metal tolerance.

The role of phytochelatins may not be restricted to the chelation of potentially deleterious ions. Zhang [126] showed that salt and heat stress could lead to a significant increase in phytochelatin concentrations in leaves and, to some extent, in roots of garlic seedlings. However, this study is somewhat contradictory, and the transcript levels of phytochelatin synthase under salt or heat stress were not elevated in the same way as under metal stress. Another report by Bhargava *et al.*[127] on the cyanobacterium, *Anabaena doliolum*, also suggested that increased concentrations and the role of phytochelatins may not be stress-specific. They showed that phytochelatins may provide some protection against UV-B radiation, suggesting an antioxidant or protein thiol-protective role. The cloning and expression of phytochelatin synthase gene from *Anabaena* into *E. coli* lead to increased tolerance to metals and also to salt, heat, UV-B and herbicide stress [128]. Upregulation of the phytochelatin synthase gene was also reported in *Arabidopsis* subjected to drought, cold and salt stress [129]. A possible role of phytochelatins in the protection of antioxidants and proteins may explain their function in tolerance to other abiotic stresses. Nonetheless, reports on the functions of phytochelatins other than in metal chelation are scarce and care should be taken to draw conclusions from potentially spurious correlations: the expression and activity of phytochelatin synthase is induced by ABA [130], but does not necessarily require the presence of metal ions. ABA is involved in plant response to abiotic stress at a very high level, with downstream effects on other plant hormones [131]. Therefore, phytochelatin synthase expression and activity may be affected by stress generally.

Despite their undoubted roles in metal stress response, some authors argued that phytochelatins are not essential for long-term tolerance and hyperaccumulation, for example, in *Sedum alfredii*[132], a plant frequently used for phytoremediation. Here, metal accumulation and sequestration was attributed to GSH, the role of which in metal chelation was recently reviewed by Jozefczak *et al.*[133]. NMR studies showed that GSH itself is a potent chelator of Cd [134]. Considering that GSH is a phytochelatin with the overall formula (γ-Glu-Cys)_n_-Gly, whereby *n* = 1, the question arises whether phytochelatin chain length is of importance for efficient chelation. The higher n is, the more ions are bound [135], but this could also be achieved by high GSH concentrations—although effective chelation would require high levels of GSH synthesis. The latter study showed that the stability constant of Cd-phytochelatin complexes is three-fold higher for *n* = 2 compared to GSH, increased a little more for *n* = 3, but did not change significantly up to *n* = 5. Hence, phytochelatins are more suitable for metal chelation then GSH. Unlike GSH, phytochelatins apparently do not form disulphides under oxidative conditions, suggesting that GSH and phytochelatins have distinct physiological functions. There is some evidence that phytochelatins may undergo S-nitrosylation [136], which could reduce the ability to chelate metals [137], but no reports exist on phytochelatin—phytochelatins disulphide redox couples, and therefore, they could be regarded as a stable form of GSH under oxidative conditions.

It is worth noting that in metal-intolerant plants, increased synthesis and accumulation of phytochelatins is not necessarily beneficial. For example, an increase in phytochelatin concentration triggered by Cu led to GSH depletion, resulting in more oxidative conditions [138]. This effect was far lower in a Cu-tolerant genotype, where Cu concentrations of 100 μM and higher resulted in increased phytochelatin concentrations without depleting GSH. Other studies also showed that GSH concentration can decrease, due to phytochelatin synthesis [139,140], but these lowered GSH concentrations were not deleterious and apparently accompanied by *de novo* GSH synthesis [140]. Heterologous expression of a wheat phytochelatin synthase gene in rice also led to increased Cd sensitivity, which was explained by GSH depletion, due to phytochelatins production [141]. By contrast, heterologous expression of phytochelatin synthase genes was related to increased metal tolerance [123]. Generally, phytochelatins are regarded as potent metal chelators, but further studies are needed to fully understand their putative role in plant stress response and to establish if this role changes upon exposure to different metal ions.

### 3.6. The Involvement of GSH in Transcriptional Control

Besides the various functions of GSH discussed earlier, there is increasing evidence that GSH is involved in the transcriptional control of various genes. An extensive study by Queval and Foyer [142] revealed that approximately 1450 genes are regulated by low GSH levels in *Arabidopsis* mutant lines. However, the majority of these genes were not regulated by low ascorbate levels or high H_2_O_2_; an overlap of about 25% was found for genes regulated by GSH and H_2_O_2_ and even less for GSH and ascorbate.

We now know that GSH can be transported into the nucleus in both plant and animal cells, where it participates in a nuclear glutathione cycle that contributes to the control of the cell cycle (reviewed by [143]). GSH may regulate the activity and function of nuclear proteins, including transcription factors by S-glutathionylation and also affect the structure of chromatin and the dynamics of chromatin condensation [144]. These processes have been far better studied in animals than in plants and need further elucidation. In the G_1_ phase of the cell cycle, when the biosynthetic activity of the cell resumes, GSH accumulates in the nucleus, and the nuclear GSH pool is far more stable than the cytoplasmic pool [145]. While the nuclear GSH can be depleted in response to oxidative stress in animal cells [146] and increased to some extent in yeast [147], very little is known about plants [148]. An example is the paper on *Arabidopsis* by Vivancos *et al.*[149], where GSH accumulation differentially affected the expression of a number of redox-related proteins. Most of the downregulated transcripts encoded heat shock proteins (HSPs) or other defensive proteins, indicating that plant cells are particularly stress sensitive at this developmental stage.

GSH may also exert its regulatory role through S-nitrosylation by NO, thus producing S-nitrosoglutathione [GSNO; 70], linking the GSH/GSSG redox state with NO signalling pathways. NO production is triggered by different stress conditions, such as cold stress [150], H_2_O_2_[151], pathogens [152], heavy metals [153] and wounding [154], and provides regulatory functions under environmental stress [155], impacting upon plant stress tolerance [156]. Over-accumulation of NO and some of it adducts can cause nitrosative stress, causing direct or indirect molecular damage, which can reduce abiotic stress tolerance [157]. GSH is a poor NO-scavenger [158], and GSNO is often regarded as a mobile source of NO and also a reactive nitrogen species (RNS) itself [159]. However, GSNO may mediate intracellular NO scavenging in a reaction that produces GSSG and NO_3_^−^[160]. GSNO concentration is regulated by GSNO reductase (GSNOR; EC 1.2.1.46), which catalyses the transnitrosylation from GSNO to protein thiols, thereby releasing GSH. A role for this enzyme in biotic and abiotic stress was proposed [161], although it is not conclusive whether a decrease [159] or an increase [161] in activity is associated with stress tolerance.

A key role of GSNO is to support the signalling functions of NO. As a donor of NO, GSNO is believed to promote the transnitrosylation of transcription factors, with downstream effects on transcription. Prominent examples are the NPR1-TGA1 system responsible for systemic resistance to pathogens in plants and the role of GSNO in enhancing the DNA-binding ability of the TGA1 transcription factor to the promoter region of the PR-1 gene in *Arabidopsis*[162]. Further evidence for this regulatory mechanism in plant defence was provided recently [163]. In contrast, blockage of the DNA binding properties of the AtMYB2 transcription factor, whose activity is induced by ABA and salt stress, occurred upon S-nitrosylation of a specific Cys-residue [164]. Wounding led to a significant increase in GSNO levels in *Arabidopsis*[165], but GSNO is also involved in salt stress tolerance (reviewed by [166]), as shown by Wang [167], by preventing electrolyte leakage from *Arabidopsis* callus.

In summary, GSH is a key ROS scavenger and major cellular redox buffer; it is a pivotal part of stress signalling pathways and has important roles in the regulation of the cell cycle.

## 4. Protein Thiols

### 4.1. Thioredoxins and Glutaredoxins

Thioredoxins (TRX) and glutaredoxins (GRX) are among the most potent protein-based protection mechanisms [168] and occur in most organisms, from bacteria (*E. coli*) to higher plants and animals. Both proteins are thiol oxidoreductases with a pair of Cys residues that provide reducing power to a variety of stress-related enzymes, like thiol peroxidase, methionine sulfoxide reductase (MSR) and arsenate reductases, or exert direct thiol-disulphide oxidoreductase activity to various protein targets. While GRX are directly reduced by GSH to produce GSSG, the reduction of TRX requires different reductases, depending on the cellular compartment. Cytosolic and mitochondrial TRX require compartment-specific NADPH-dependent TRX reductases [169], and plastid TRX are reduced by ferredoxin/TRX reductase [170].

The number of TRX genes may vary between plant species from 60 to 70 in rice and *Arabidopsis* to 11 in sorghum, and the number of genes expressing a TRX family may also differ from species to species [171]. It has been suggested that this enrichment of TRX genes in the genome is the reason why their transcriptional control varies substantially, depending on species and stress factor. Abiotic stresses elevate TRX either on the gene and/or protein level. A proteomics study showed that putative TRX and GRX genes were upregulated in rice under Cu stress [56]. The fold increase was also substantially higher in a Cu tolerant line than in a sensitive line, and a similar correlation was reported for salt-resistant and salt-sensitive barley genotypes [172]. In a genome-wide study of rice, Nuruzzaman *et al.*[171] showed significant differences in the TRX gene expression in conditions of biotic and abiotic stress. Interestingly, cold stress seemed to downregulate most TRX genes, but drought stress led to upregulation, at least at the earlier stages of the stress treatment. However, it should not be accepted *a priori* that a particular stress factor will necessarily lead to the upregulation of a particular TRX gene. TRX and GRX have various functions, such as chaperone activity reported for plastid TRX in tobacco [173]. As shown in *Arabidopsis*, the family of tetratricopeptide thioredoxin-like (TTL) proteins are essential for salt and osmotic stress tolerance, and mutations of their genes altered both stress response and development [174]. Despite the *C*-terminal thioredoxin-like domain, their activity is related to co-chaperon interactions with Hsp90 and Hsp70 [175]. Other proteins related to TRX or GRX may also exhibit chaperone-like functions and confer stress tolerance. Thioredoxin reductase type C (NTRC) in *Arabidopsis* underwent heat-shock induced oligomerization and exhibited holdase or foldase chaperone functions to provide thermotolerance [176]. It was found that the full-length cDNA of NTRC encoded TRX motifs additionally to the TR motif. It seems that NTRC is a good example of a protective protein with multiple functions and different roles, depending on the available substrate, such as reduction of protein disulphide bonds under oxidising conditions or chaperone functions to non-Cys proteins. As proposed by Chae *et al.*[176], this is an example of “functional promiscuity” or acquisition of a novel function without losing the primary one. In contrast, the TTL protein family may be an example of the opposite function. Overall, the NADPH-dependent TRX and GRX systems were shown to belong to the most prominent players in stress protection among both thiol-containing and non-thiol proteins.

### 4.2. Glutathionylation

Cys residues in macromolecules are extremely vulnerable to oxidative modification, such as those associated with ROS production in adverse environmental conditions. Cys residues undergo a three-step oxidation to cysteine sulphenic acid (Cys-SOH) that is reversible, then irreversible oxidation to cysteine sulphinic acid (Cys-SO_2_H) and cysteine sulfonic acid (Cys-SO_3_H). The irreversibility of the protein-Cys-SO_2_H has been questioned [177], and in *Arabidopsis*, sulphiredoxins are believed to have sulphinic acid reductase activity [178]. Such proteins lose their native conformation and activity and are further processed to be degraded (summarised in [179]). Glutathionylation of the first product, protein-Cys-SOH, could be produced through reaction with GSSG or be enzymatically catalysed, leading to temporary protection of Cys-residues, a reversible protection mechanism. Cys can be reformed through reaction of the residues with GRX, which strongly depends on GSH concentration and GR activity [180]. Oxidative damage was also reported for Met residues. Extensive glutathionylation and increased GRX expression are likely to enhance stress tolerance. However, neither the exact mechanism nor the physiological relevance of protein glutathionylation in plants is established to date. Most of the results were obtained *in vitro*, and the prevalent understanding is that the spontaneous glutathionylation by GSSG is far less likely *in vivo*[181]. Most of the recent data on glutathionylation of proteins in relation to stress tolerance relate to deglutathionylation and the involvement of GRX.

**Figure 2:**
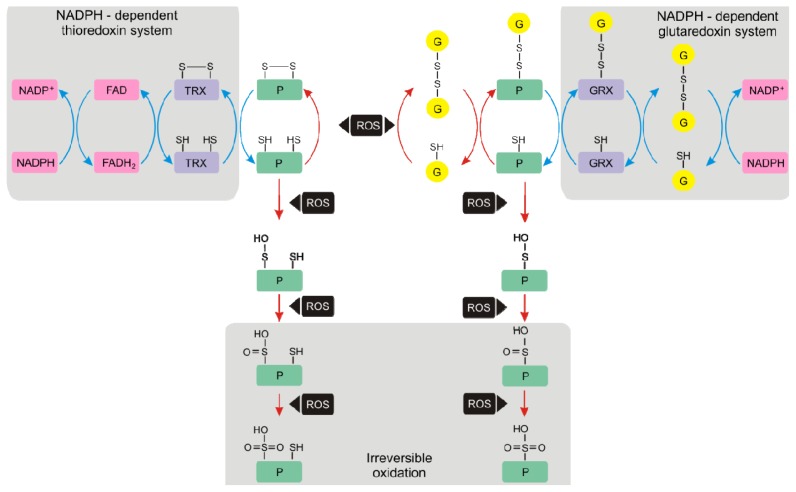
Thiol-disulphide cycle, proposed to contribute to the biochemical mechanisms that confer desiccation tolerance, involving glutathionylation of proteins, GRX and TRX. This figure is reproduced from Colville and Kranner [14], with the permission of Plant Growth Regulation. Desiccation tolerant organisms can lose more than 90% of their water without dying and revive upon rehydration, and extreme fluctuations in water content are accompanied by equally extreme changes in cellular redox state, associated with an increase in ROS levels. Red arrows: processes that may occur predominantly during desiccation; blue arrows: processes that occur primarily during rehydration. The thiol (-SH) groups of redox-active Cys residues in proteins (P; green) and glutathione (G; yellow) are susceptible to oxidation, and in the dry state, a shift from thiols to the disulphide forms occurs as the probability for enzymatic reduction decreases with progressive water loss. Glutathionylation protects protein Cys residues from further oxidation to sulphenic (PSOH), sulphinic (PSO_2_H) and sulphonic (PSO_3_H) acids. Sulphenic acid can be reduced by glutathione, whereas sulphonic and sulphinic acid formation are thought to be irreversible. Glutathionylation may occur through several mechanisms, for example, through reactions between a protein thiol (PSH) and GSSG, forming protein-bound glutathione (PSSG). This will occur under conditions of severe oxidative stress when GSSG accumulates. Other mechanisms include reactions of protein thiyl radicals or sulphenic acid intermediates with GSH; glutaredoxins (GRX) can also catalyse the reaction between GS^•^ and PSH to produce PSSG (not shown), although GRXs normally act as reductants in de-glutathionylation reactions. Upon rehydration, the reduction of protein disulphides can be catalysed by thioredoxin (TRX), which is subsequently reduced by TRX reductases (NADPH-dependent thioredoxin system). The NADPH-dependent thioredoxin system operates in the cytosol and mitochondria, where TRX reductases utilise electrons supplied by NADPH, which are transferred to TRX disulphide via flavoproteins (FAD). In chloroplasts, TRX reductases may use ferredoxin as an electron donor. Moreover, upon rehydration GRXs can reduce PSSG, leading to the formation of a mixed disulphide between GSSG and GRX. This is reduced by GSH, and the resulting GSSG is reduced by GR (NADPH-dependent glutaredoxin system). The active site of GRXs can either have one (monothiol) or two (dithiol) catalytic Cys residues; only the monothiol mechanism for PSSG reduction is shown.

Colville and Kranner [14] recently reviewed the current literature on desiccation tolerant organisms, in which extreme fluctuations in water content during wetting and drying cycles are accompanied by equally extreme changes in their cellular redox state. In these organisms, protein glutathionylation is a likely contributor to protection mechanisms that confer desiccation tolerance. Upon rehydration, de-glutathionylation was suggested to be catalysed by glutaredoxins and protein disulphide bonds to be reduced through NADPH-dependent thioredoxins (Figure 2). This thiol-disulphide cycle appears to have specific importance for desiccation tolerance and may play a more general role in plant stress tolerance.

Protein glutathionylation can be regarded a post-translational regulatory mechanism with significant effects on the activity of various enzymes and transcription factors. Among the best-characterised stress-related proteins that undergo glutathionylation are the members of the annexin superfamily [182]. Annexins are membrane-associated, Ca^2+^-dependent proteins with putative enzymatic activity involved in signal transduction. Differential upregulation of annexin-encoding genes was related to various stress treatments [183]. Not surprisingly, glutathionylation of annexin 1 in *Arabidopsis* altered its Ca^2+^-binding affinity, which will lead to decreased function or loss of function [183]. Protein function can be altered by glutathionylation, for example, enolase and 6-phosphogluconolactase can be inhibited, whereas apoptosis signal-regulated kinase 1 can be activated [184]. As for annexin 1, it was proposed that glutathionylation may have a function in ROS sensing or in shifting signalling pathways—from Ca^2+^-mediated to ROS-mediated [185]. Several studies support the possibility that the activity of enzymes of the Calvin cycle and glycolysis can decrease, due to glutathionylation under oxidative stress, which may be an important regulatory mechanism of plant carbohydrate metabolism [15]. However, despite these recent findings, protein glutathionylation in plants still remains one of the poorly studied aspects in abiotic stress response.

## 5. Conclusions and Future Perspectives

Areas of arable land available to an ever-increasing human population are on the decrease, and more stress tolerant crops could support future farming. Independent of the strategy used to improve crop tolerance—either by marker-assisted selection, recruitment of new potential crop species or gene transformation approaches, an in-depth understanding of the mechanisms of stress tolerance is required. Plant thiols are apparently involved in plant response to almost all stress factors, and their accumulation, redox status and regulation are key to plant stress tolerance. Minor changes in gene expression or redox state could define the difference between tolerant and susceptible genotypes or species. Despite the recent progress in the research into plant thiols, there are still many open questions. For example, the regulation of thiol biosynthesis, the maintenance of their redox state and the interconnections between different biochemical pathways are not fully understood. Nonetheless, plant thiols and plant sulphur metabolism generally deserves attention with a view to improving agricultural practice in stressful environments.

## Figures and Tables

**Table 1:** Selected reports on glutathione-S-transferase (GST) overexpression and/or heterologous expression that enhanced abiotic stress tolerance in plants. All examples include mutational overexpression of endogenous GST or transgenesis of GSTs from wild species and basal eukaryotes with no economic significance into commercial species and model plants.

Type of stress	Notes	Species	Reference
Cd	GST gene from *Trichoderma virens*	Tobacco	[104]
Cold	GST gene from *Choristoneura fumiferana*	*Arabidopsis*	[105]
Drought	Expression of GST gene from *Prosopis juliflora*	Tobacco	[106]
Drought and salt	GST gene from *Glycine soja*	Tobacco	[101]
Heavy metals	Human GST and CYP2E1 genes	Alfalfa	[107]
Herbicide	Overexpression of GST	Rice	[108]
Salt	Overexpression of GST	*Arabidopsis*	[102]
Salt	GST gene from *Salicornia brachiata*	Tobacco	[109]

**Table 2:** Selected publications from 2012 on plant heavy metal tolerance conferred by phytochelatins. All papers relate to endogenous phytochelatin synthesis except for those with symbols.

Metal tolerance	Plant species	Reference
As	*Wolffia globosa*	[113]
	*Oryza sativa*	[114]
	*Oryza sativa*	[115]
	*Pteris vittata*	[116]
Cd	*Brassica juncea*	[117]
	*Arabidopsis thaliana*	[118]*
	*Populus nigra*	[119]
	*Oenothera odorata*	[120]
	*Triticum aestivum*	[121]
	*Linum usitatissimum*	[122]
Cd, As	*Nicotiana tabacum*	[123]*
Cd, Cu, As, Zn	*Arabidopsis thaliana*	[124]^
Mn	*Vitis vinifera*	[125]

* heterologous expression of phytochelatin synthase gene;

^ expression of yeast phytochelatin transporters.

## Abbreviations

ABA: abscisic acid
ASC: ascorbate
ASCPx: ascorbate peroxidase
Cys: cysteine
CySS: cystine, cysteine-disulphide
DHA: dehydroascorbate
DHAR: dehydroascorbate reductase
EGSSG/2 GSH: half-cell reduction potential of the glutathione disulphide-glutathione redox couple
GPx: glutathione peroxidase
GR: glutathione reductase
GRX: glutaredoxin
GSH: glutathione
GSNO: S-nitrosoglutathione
GSSG: glutathione disulphide
GST: glutathione-S-transferase
hGSH: homoglutathione
hGSSG: homoglutathione disulphide
HSP: heat shock protein
MDHAR: monodehydroascorbate reductase
Met: methionine
MSR: methionine sulfoxide reductase
NTRC: thioredoxin reductase type C
PC: phytochelatins
PCS: phytochelatin synthetase
PRX: thiol peroxidase
RNS: reactive nitrogen species
ROS: reactive oxygen species
SAM: S-adenosyl methionine
SAT: serine acetyltransferase
SOD: superoxide dismutase
TRX: thioredoxin
TTL: tetratricopeptide thioredoxin-like
